# Permeabilities and Mechanical Properties of Hardened Cement Pastes Modified with Sodium Laurate and Nano Silica

**DOI:** 10.3390/ma13214867

**Published:** 2020-10-30

**Authors:** Fajun Wang, Sheng Lei

**Affiliations:** 1 School of Materials Science and Engineering, Nanchang Hangkong University, Nanchang 330063, China; jjbxsjz@foxmail.com; 2 School of Materials Engineering, Jiangsu University of Technology, Changzhou 213001, China

**Keywords:** hardened cement paste, hydrophobicity, nano silica, sodium laurate, compressive strength, water absorption

## Abstract

In this work, a method of imparting hydrophobicity and high strength to hardened cement paste (HCP) is proposed. Sodium laurate (SL) was used as a hydrophobic modifier and nano silica (NS) as a pozzolan. The HCP was modified by SL and NS simultaneously. HCP modified with different contents of SL and NS was prepared. Surface wettability, micro-structures, chemical composition, and organic structure were systematically studied using contact angle (CA) measurement, scanning electron microscope (SEM) observation, X-ray photoelectron spectroscopy (XPS), and attenuated total reflection Fourier transform infrared spectroscopy (ATR-FTIR), respectively. The surface CA of the sample is 138.5° and has high hydrophobicity. Compared with the reference sample, the water absorption of the modified sample reduced by 96.55%, while the compressive strength only reduced by 6.91%. Therefore, using hydrophobic modifier and reinforcing agent as cement admixture is an effective method to endow concrete with hydrophobicity and high strength at the same time.

## 1. Introduction

Most of the corrosion effects on concrete during service are related to water [1,2,3]. The corrosion of concrete is essentially the corrosion of hardened cement paste (HCP). Silicate-based HCP is composed of hydrated calcium silicate (CSH), portlandite (CH), ettringite (AFt), unhydrated cement powder (C), and a large number of pores [2]. The surface of HCP contains a large number of hydroxyl groups and is hydrophilic. Therefore, water can easily penetrate HCP [4,5,6]. When concrete is in service, it is unavoidable to come into contact with water, rain, snow, and other water sources in rivers and lakes. For example, in winter, deicing salts on road surfaces usually contain Cl^−^ [7,8,9]. Rainfall contains acid substance and lakes may contain SO_4_^2−^. Seawater contains a variety of aggressive ions, such as Mg^2+^, Cl^−^, and so on. These substances usually enter the interior of the concrete through the penetration of water solution, thereby causing corrosion of the concrete. Therefore, it is necessary to reduce the water absorption rate of concrete and improve the permeability resistance of water in concrete to improve the durability of concrete [10,11,12].

Hydrophobic modification method is an effective method to improve the impermeability of concrete and has received extensive attention in recent years [5,9,11]. The most commonly used hydrophobic modifiers are silane reagents, such as n-octyl triethoxy silane and isobutyl triethoxy silane [6,10]. On the surface of the solidified concrete, the surface hydrophobicity is obtained by silane impregnation, brushing, or spraying. Silanes can also be added to newly-prepared concrete mixtures. They exhibit a bulk hydrophobicity (both the surface and the inside are hydrophobic) after curing [6,13]. However, silane reagents are very expensive, which limits the large-scale use of silane in concrete. Another kind of hydrophobic modifier for concrete are stearic acid-based reagents, including stearic acid, sodium stearate, calcium stearate, etc. [12,14,15]. These reagents are cheap but they are solid at room temperature and insoluble in water. Therefore, it is difficult to mix them with other components in concrete, which limits their use.

When a hydrophobic agent is added to concrete as an admixture, it often has an adverse effect on the strength of the concrete. For example, Song et al. prepared superhydrophobic concrete using fluorosilane as an admixture. Song used fluorosilane as an additive to prepare superhydrophobic concrete. Superhydrophobic concrete shows excellent corrosion resistance to chloride ingress and anti-icing performance. However, the compressive strength of concrete is reduced by 40%. The reduction in strength can be explained, as the addition of silane hinders the full hydration of cement. The decrease in concrete strength will result in the failure of concrete components to meet the strength standard of engineering application. Therefore, it is urgent to develop modified materials that can give concrete hydrophobicity without reducing the mechanical strength of concrete. It is well known that some pozzolanic active materials, such as nano silica, [16] silica fume, [17] granulated blast furnace slag, [18] and fly ash [19], can significantly improve the strength of concrete. Therefore, if the hydrophobic modifier and active powder are added to the concrete at the same time, it is possible to prepare concrete material with both hydrophobicity and high strength.

In the present work, in order to obtain HCP with both hydrophobicity and higher strength, sodium laurate (SL) and nano silica (NS) are used as cement admixtures to synergistically modify the HCP. SL dissolves easily in water at room temperature, which ensures that LS can be uniformly mixed with the concrete components in water. SL imparts superhydrophobicity to concrete but causes a significant decrease in HCP strength, while the NS improves the strength of HCP. As a result of the synergistic modification, the HCP has both hydrophobicity and high strength (>40 MPa). The modified HCP exhibited a surface contact angle (CA) of 138.5°. Additionally, compared with the reference sample, the compressive strength of the modified sample decreased by only 6.91%, while the water absorption decreased greatly by 96.55%. This work provides a new idea and method for realizing the hydrophobicity and high strength of concrete.

## 2. Materials and Method

### 2.1. Materials

Ordinary Portland cement (CEM I 42.5 N) was purchased from Anhui Conch Cement Co., Ltd. (Hefei, China) Sodium laurate (SL, purity > 98%) was purchased from Shanghai Chemical Reagent Co., Ltd. (Shanghai, China). Nano silica (NS, M5, 20 nm, purity > 99%) was purchased from Cabot Chemical Co., Ltd. (Shanghai, China).

### 2.2. Preparation of Cement Paste Modified with SL and NS

A series of hardened cement pastes (HCP) modified by SL and/or NS were prepared according to the formula shown in Table 1. The HCP samples modified with different concentrations of SL were named as HCPSLx, where x refers to the mass percentage of cement replaced by SL. Similarly, the HCP samples modified with different concentrations of NS were named as HCPNSx. For the HCP modified with SL and NS simultaneously, the sample were name as HCPSLxNS. In the HCPSLxNS samples, the content of NS was fixed at 1.0 wt.%, while the content of SL was varied from 0.25 wt.% to 1.25 wt.% in increments of 0.25 wt.%. SL was dissolved in tap water before mixing. The dry powders of cement and NS were pre-mixed in a ball mill for 30 min. Then, the mixed dry powder was gradually added to SL aqueous solution under mechanical stirring. After the dry powders were added, the mixture was further mixed for 5 min under high-speed mechanical mixing to ensure that the cement powder and water were fully mixed. The mixed slurry was formed in a mold. After 24 h, the solidified cement paste was demolded. The HCP samples were cured to different ages (3, 7, 14, and 28 d) in a standard curing box before use.

### 2.3. Characterization

#### 2.3.1. FE-SEM, XPS, and FTIR Measurement

The surface micro-structures of the various HCP samples were observed using a field emission scanning electron microscope (FE-SEM, FEI Quanta F150, Waltham, MA, USA). The surface chemical elements and their binding states were measured by X-ray photoelectron spectroscopy (XPS, Thermofisher, Escalab 250Xi+, Waltham, MA, USA). The structure of organic groups on the surface of the samples was measured using attenuated total reflection Fourier infrared spectroscopy (ATR-FTIR, Bruker, Vertex 70, Karlsruhe, Germany). After 28 days of curing, the SEM, XPS, and FTIR of the samples were tested.

#### 2.3.2. Surface Wettability Measurement

The water contact angles (CA) of various HCP samples were tested using an DSA100 optical goniometer (Krüss, Hamburg, Germany). The test process was carried out at room temperature, the test liquid used was deionized water, and the volume of water droplets used was fixed at 10 microliters. The CA was measured at 6 different places on the surface of the HCP and the average value was taken as the reported value in this work. After 28 days of curing, the surface CAs of the samples were tested.

#### 2.3.3. Measurement of Flexural Strength and Compressive Strength

The flexural strength and compressive strength of various HCP samples were measured according to the Chinese Standard [JG/T 1169-2005] [14]. Five prism samples with dimensions of 160 mm × 40 mm × 40 mm were used to test the flexural strength and the average value was taken as the final reported value. The specimen after flexural strength test was used for the compressive strength test. Five samples were used to test the compressive strength and the average value was taken as the final reported value. The mechanical strength of the samples was tested after curing to different ages (namely, 3, 7, 14, and 28 d).

#### 2.3.4. Measurement of Water Absorption

The water absorption of various HCP samples was measured using the method described in ISO 15148:2002(E) [20]. After 28 days of curing, the water absorption of the samples was tested.

## 3. Results and Discussion

### 3.1. Wettability

**Figure 1:**
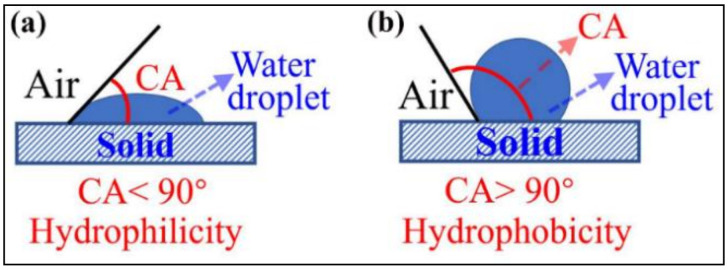
Illustration of the surface wettability of a solid. (**a**) Hydrophilicity and (**b**) hydrophobicity.

**Figure 2:**
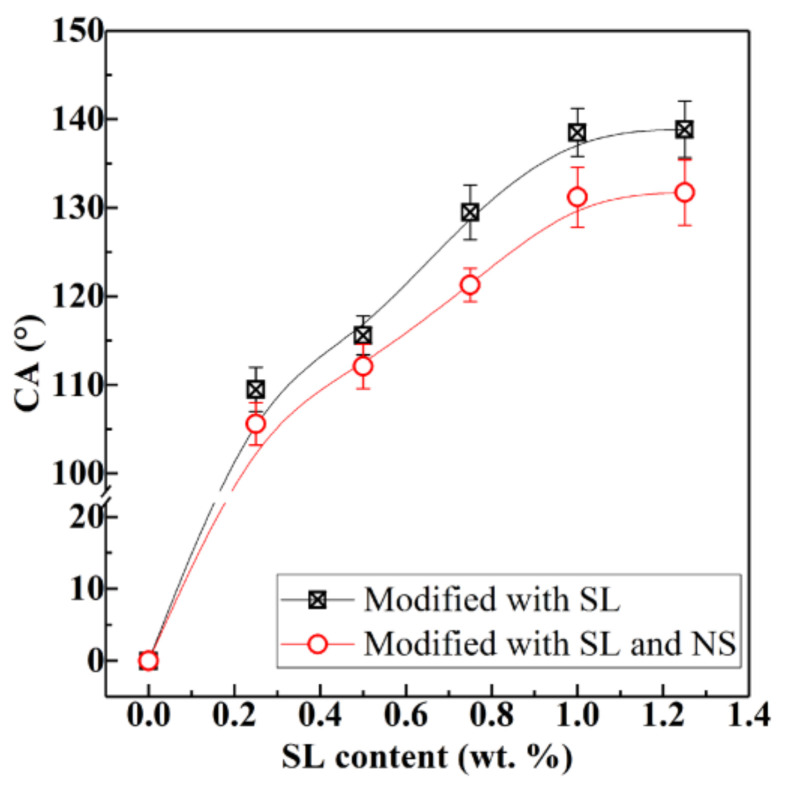
Influence of SL concentration on the surface contact angle (CA) of pure hardened cement pastes (HCPs) and NS-modified HCPs; the content of NS in the HCP is 0 wt.% (HCP) and 1.0 wt.% (NS-modified HCPs or HCPNS), respectively.

**Figure 3:**
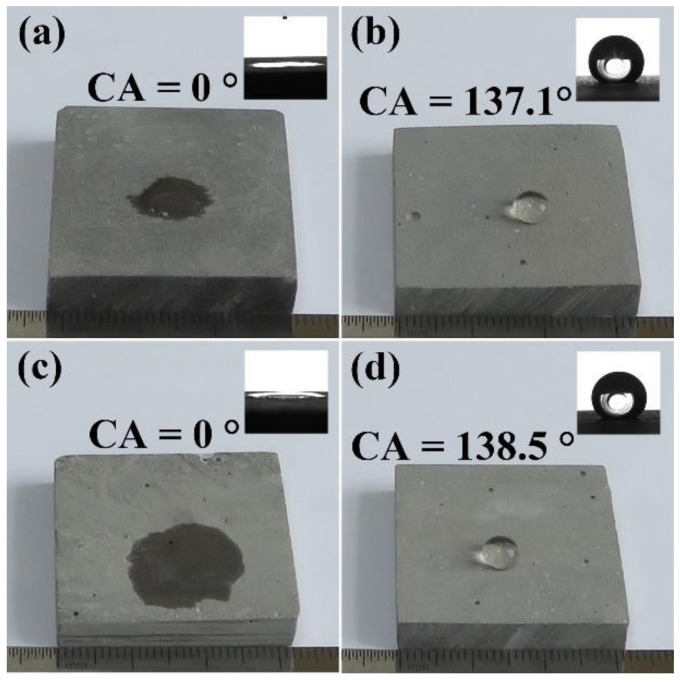
Photos of water droplets on the surfaces of different HCPs: (**a**) pure HCP; (**b**) 1.0 wt.% SL-modified HCP; (**c**) 1.0 wt.% NS-modified HCP; (**d**) 1.0 wt.% SL and 1.0 wt.% NS co-modified HCP.

The wettability of solid surface is expressed by surface CA, as shown in Figure 1. Water spreads easily on the hydrophilic surface and the CA is less than 90° (see Figure 1a). The water is difficult to spread on the hydrophobic surface, shrinks into a ball, and the CA is greater than 90° (see Figure 1b). Figure 2 shows the effect of SL addition on the surface CA of pure HCP and HCPNS. The HCP is a hydrophilic material and its surface CA is 0°. Similarly, the HCPNS is also hydrophilic and its surface CA is also 0°. With the increase in SL content, the surface CAs of both the HCP and the HCPNS increase. When the content of SL is 1.0 wt.%, the CAs of HCP and HCPNS are 138.8° and 137.5°, respectively. The surface CAs of both HCP and HCPNS increase a little with further increasing SL content. Figure 3 shows the wettability of water droplets on the solid surface after adding water droplets to the surface of different HCPs. Water droplets completely infiltrate on the surface of HCP and nano silica-modified HCP, as shown in Figure 3a,c. The pozzolanic reaction between NS and portlandite, the hydration product of alite and belite, resulted in the formation of calcium silicate hydrate (C-S-H) gel. C-S-H is the main gel product in HCP and has hydrophilicity. Therefore, the wettability of HCP will not change after the introduction of NS into Portland cement. Similarly, sodium laurate can react with calcium hydroxide to produce water-insoluble calcium laurate:

(R1) $$CH_{3}{\left(CH_{2}\right)}_{10}COONa+Ca{\left(OH\right)}_{2}\to {\left[CH_{3}{\left(CH_{2}\right)}_{10}COO\right]}_{2}Ca+NaOH$$

Calcium laurate is hydrophobic because of the long-chain alkyl groups attached to the calcium atom. It has been reported that calcium stearate, zinc stearate, manganese stearate, and other stearate salts were used to prepare hydrophobic/superhydrophobic materials. For example, Li et al. used calcium stearate (CaSt) as an alternative admixture to reduce the water absorption of slag cement [12]. The results show that when the addition amount of CaSt is 4%, the water absorption of the sample can be significantly reduced. Li et al. prepared a zinc stearate superhydrophobic coating with high adhesion by electrodeposition [21]. The coating exhibited a high water CA of 164.8 ± 0.6° and high adhesive properties simultaneously. Parsaie et al. fabricated a superoleophilic/superhydrophobic sponge coated with phenol-formaldehyde resin and magnesium stearate for oil/water separation [22]. It has been confirmed in previous literature that some stearates, especially calcium stearate, magnesium stearate, zinc stearate, and copper stearate coatings, possessed superhydrophobicity [21,22,23]. The superhydrophobicity of these insoluble stearate coatings can be attributed to the combination of the microscopic rough structure formed by the accumulation of stearate particles and the non-polar linear alkyl group containing 18 carbon atoms.

**Figure 4:**
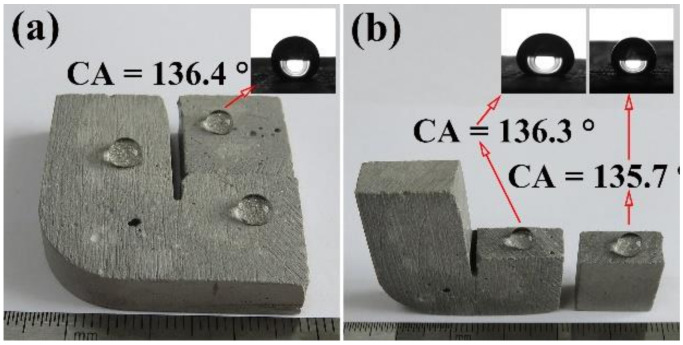
Photos of water droplets on different surfaces of HCPSL1.0NS1.0: (**a**) as-prepared surface; (**b**) newly exposed surface after being broken.

Images of water droplets on the surface of four typical samples, namely the HCP (HCP), the SL-modified HCP (HCPSL), the NS-modified HCP (HCPNS), and the SL and NS co-modified HCP (HCPSLNS), are shown in Figure 3a–d. When the water drops were added to the surface of HCP and HCPNS, the water droplets spread rapidly on the surfaces of the samples, indicating that the two samples are hydrophilic (Figure 3a,c). When water droplets were dripped onto the surfaces of samples 3 and 4, the water droplets sat on the surface of the sample and were approximately spherical, with CAs of 137.1° and 138.5°, respectively, indicating that these two surfaces are hydrophobic (Figure 3b,d). The hardened cement paste modified by sodium laurate and nano silica not only has hydrophobicity on the surface but also has hydrophobicity inside (see Figure 4a,b).

### 3.2. SEM

**Figure 5:**
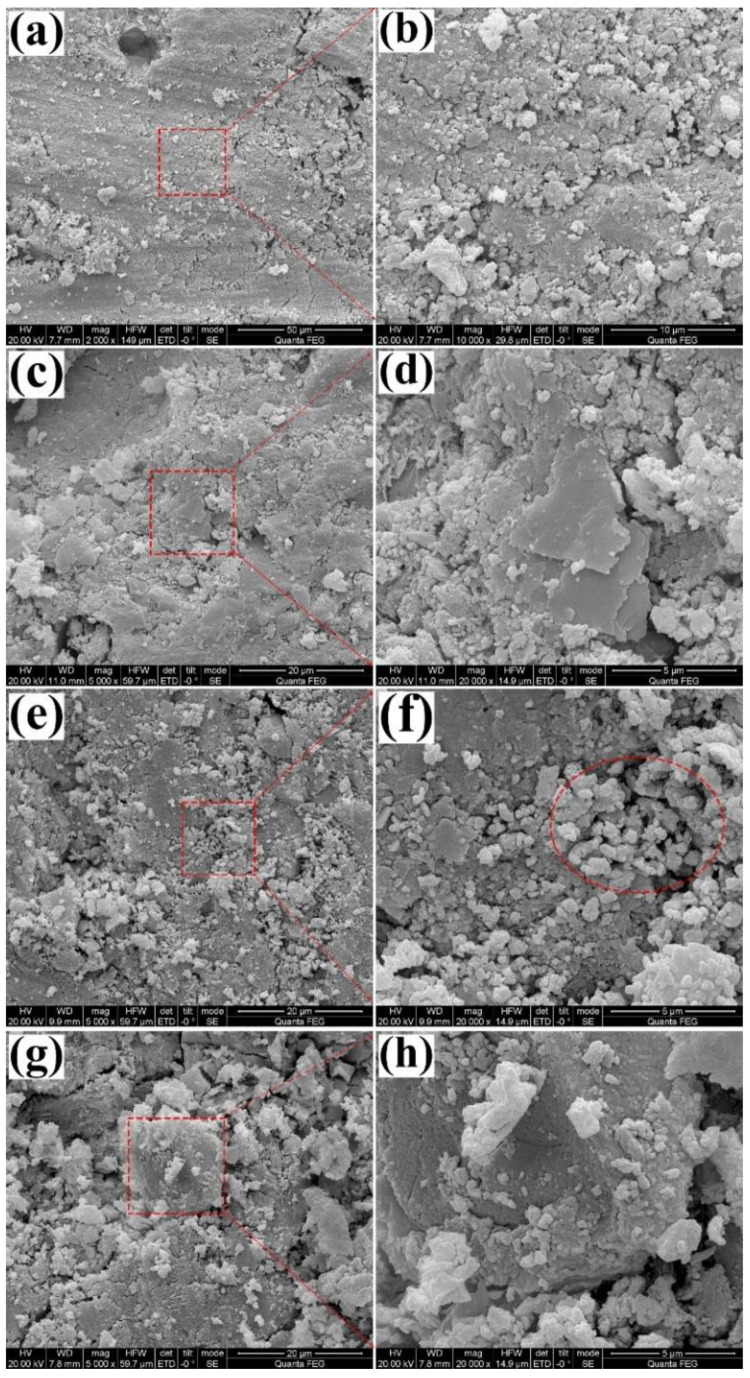
Surface field emission scanning electron microscope (FE-SEM) images of different HCPs at different magnifications: (**a**,**b**) pure HCP; (**c**,**d**) HCP modified with 1.0 wt.% SL; (**e**,**f**) HCP modified with 1.0 wt.% NS; (**g**,**h**) HCP modified with 1.0 wt.% SL and 1.0 wt.% NS simultaneously.

The SEM micro-structures of various HCP samples are shown in Figure 5. The pure HCP is mainly composed of C-S-H gel, calcium hydroxide (CH), ettringite (AFt), residual cement powder, and pores. Comparing the surface SEM images of the HCP and the HCPSL, one can see that there was no obvious difference in the surface microstructures of the two samples. Both of them were composed of irregularly shaped and closely packed fine particles of different sizes. Many agglomerates of fine particles can be found on the surface of the HCPNS, which may be caused by the agglomeration of nano silica particles (see the highlighted section in Figure 5f). Nano silica plays two roles in the HCP. The first role is to fill in the gaps of the HCP, making the HCP more compact. Another effect is to consume CH through the pozzolanic reaction and promote the formation of C-S-H. Both of them are beneficial to improve the strength of the HCP and reduce the water absorption of the HCP. However, the dispersibility of nano silica in cement paste is poor and its advantages are difficult to fully utilize. Especially when the concentration of the NS is large, it even causes deterioration of the performance of the HCP. Therefore, the content of NS used in this work is only 1 wt.%. It is also observed that the pores in HCPSLNS are obviously larger than those in the other three samples (CHP, HCPSL, and HCPNS). There are many pores in the HCPSLNS, which can be explained by the formation of calcium laurate covering the surface of CH, which increases the interfacial resistance between CH and NS. Therefore, the pozzolanic reaction of nano silica is blocked, which is not conducive to the densification of the gel system, resulting in more pores.

### 3.3. XPS and FT-IR

**Figure 6:**
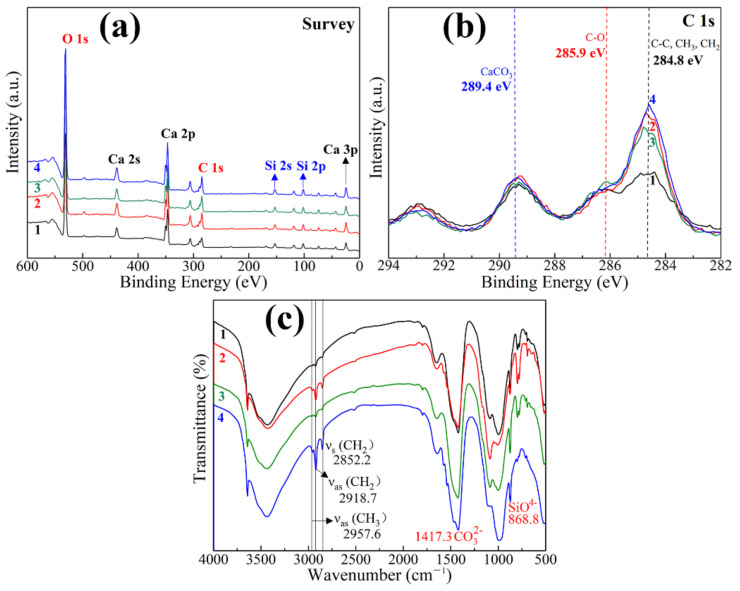
(**a**) X-ray photoelectron spectroscopy (XPS) survey spectra of various sample surface; (**b**) high resolution XPS spectra in C 1 s region of various sample surface; (**c**) attenuated total reflection Fourier transform infrared spectroscopy (ATR-FTIR) spectra of various sample surfaces. Curves 1, 2, 3, and 4 correspond to the following four samples, respectively: 1, Pure HCP; 2, HCP modified with 1.0 wt.% SL; 3, HCP modified with 1.0 wt.% NS; 4, HCP modified with 1.0 wt.% SL and 1.0 wt.% NS simultaneously.

The wettability of solid surface is determined by two factors: the first is surface roughness and the second is surface energy. As the roughness of solid surface increases, the hydrophobic surface becomes more hydrophobic and the hydrophilic surface becomes more hydrophilic. The polarity of the sample surface was tested and analyzed using XPS and FTIR. The XPS survey spectra of different sample surfaces are shown in Figure 6a. It can be observed that the samples of HCP, HCPSL, HCPNS, and HCPSLNS contain elements of calcium, silicon, carbon, and oxygen. Calcium, silicon, and oxygen can be attributed to the silicon and calcium compounds in Portland cement hydration products. In samples HCP and HCPNS, the carbon element is derived from carbon adsorption and/or carbonization, while in samples HCPSL and HCPSLNS, the carbon element can be attributed to the carbon derived from SL and carbonization. Figure 5b shows the high resolution XPS spectra of different samples at C 1 s region. The peak located at 284.8 eV was assigned to the non-polar carbon (-CH_3_, -CH_2_, C-C) on the sample surfaces. The peak centered at 285.9 eV was ascribed the polar carbon (C-O) on the surfaces [24], while the peak located at 289.4 eV could be attributed to the formation of calcium carbonate [24]. Low surface energy is necessary for the hydrophobicity of the solid surface. The more non-polar carbon content on the surface of the material, the lower the surface energy of the material. One can see that the intensity of the non-polar carbon contained in HCPSL and HCPSLNS is significantly higher than that in HCP and HCPNS (Figure 6b). Therefore, the surfaces of HCPSL and HCPSLNS exhibit hydrophobicity, while the surfaces of HCP and HCPNS exhibit hydrophilicity. The ATR-FTIR spectra of various sample surface are shown in Figure 6c. It was observed that the samples show almost identical infrared spectral curves (Figure 6c). The peak located at 1417.3 cm^−1^ was ascribed to the stretching vibration of CO_3_^2−^ in carbon carbonate [25]. Bands in the range 860~1000 cm^−1^ are ascribed to the linking tetrahedra of SiO^4-^. In the ATR-FTIR spectra of HCPSL and HCPSLNS, the absorption peaks of symmetric stretching vibration (*ν_s_*) and asymmetric stretching vibration (*ν_as_*) of the -CH_2_ group related to non-polar bonds are located at 2852.2 cm^−1^ and 2918.7 cm^−1^, respectively [26]. The absorption peak intensity of non-polar groups in HCPSL and HCPSLNS is significantly higher than that of HCP and CHPNS, indicating that the surface energy of these two samples is lower.

**Figure 7:**
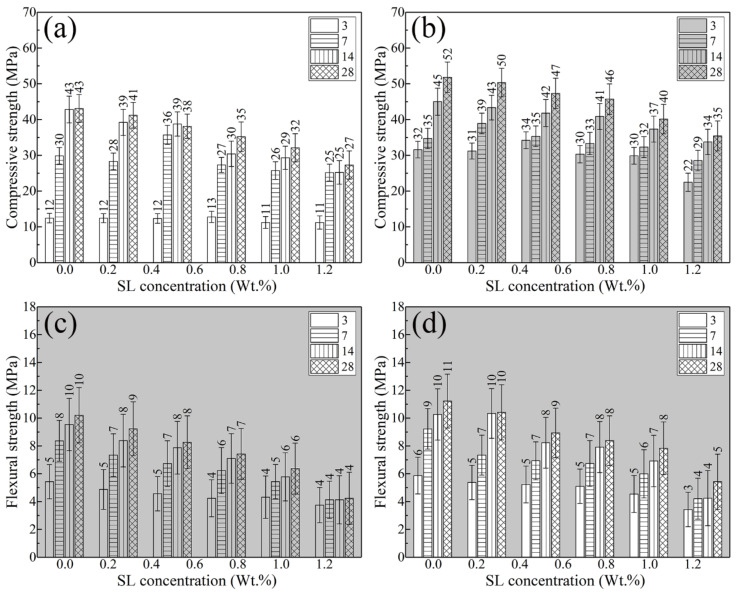
(**a**) Effect of SL concentration on the compressive strength of HCP; (**b**) effect of SL concentration on the compressive strength of HCP containing 1 wt.% NS; (**c**) effect of SL concentration on the flexural strength of HCP; (**d**) effect of SL concentration on the flexural strength of HCP containing 1 wt.% NS.

The compressive strength and flexural strength test results of different samples are listed in Figure 7. One can see that the compressive strength and flexural strength of all samples increased with the increase in curing age. The compressive strength and flexural strength of the HCP decreased with the increase in SL concentration. The introduction of 1% nano silica is beneficial to improve the compressive strength and flexural strength of the HCP. At 28 days of age, the compressive strength and flexural strength of the pure HCP were 43.12 and 10.21 MPa, respectively. When 1.0 wt.% SL was introduced, the compressive strength of HCPSL1.0 was 32.12 MPa and the reduction rate was 25.51% compared with the reference sample. The flexural strength of HCPSL1.0 was 6.37 MPa and the reduction rate was 37.61% compared with the reference sample. When 1.0 wt.% SL and 1.0% NS were introduced at the same time, the compressive strength and flexural strength of the samples (HCPSL1.0NS) were 40.14 and 7.84 MPa, respectively. Compared with the reference sample, the reduction rates were 6.91% and 23.21%, respectively. The strength test results show that the introduction of SL and NS into the HCP can endow the HCP with bulk hydrophobicity and maintain high mechanical strength of the CHP.

**Figure 8:**
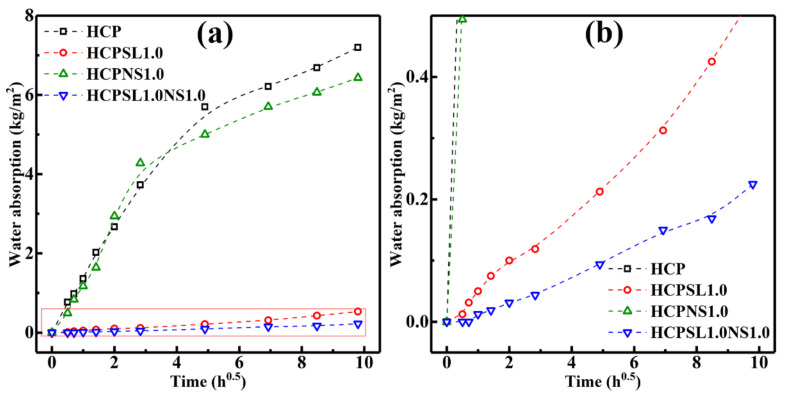
Water absorption measurement of the four kinds of HCPs; (**b**) is a partial enlargement of (**a**).

The water absorption amount (wt.%) of the sample was calculated by Equation (1):

Water absorption (wt.%) = (*w*_1_ − *w*_0_)/_w0_ (1)

where *w*_0_ is the initial weight of the sample; *w*_1_ is the weight of the sample at saturated water absorption. The water absorption test results of different samples are shown in Figure 8. The relation between capillary water absorption rate and water absorption time is expressed by Equation (2): [20]

(2) $$\Delta W=A\times \sqrt{t}$$

In Equation (2), *W* is capillary water absorption amount per unit area of sample, *t* is the immersion time, and *A* is the capillary suction coefficient. The slope of the line obtained from the best linear fitting results of the four curves in Figure 8 was the coefficient of the capillary water absorption. The results of water absorption and capillary water adsorption coefficient of various HCP samples were summarized in Table 2. One can see that the water absorption and the capillary water adsorption coefficient of the HCPSL1.0NS1.0 reduce by 96.37% and 96.55%, respectively, in comparison with the HCP. Therefore, the use of SL and NS to synergistically modify the HCP is an effective method to reduce the water absorption of the HCP.

## 4. Conclusions

Hydrophobic and high-strength HCP was prepared by using water-soluble SL as a cement hydrophobic admixture and NS as a cement reinforcer. Sodium laurate is cheap and easily soluble in water at room temperature, so it is easy to mix with cement powder in water uniformly. When the amount of SL is 1.0 wt.% and the amount of NS is 1.0 wt.%, the surface CA of the HCP is 138.5°. The compressive strength, flexural strength, and water absorption of the HCP modified by LS and NS are 40.14 MPa, 7.84 MPa, and 0.36% respectively. Compared with the unmodified HCP, the compressive strength, flexural strength, and water absorption of modified HCP were reduced by 7%, 23% and 97%, respectively. The durability of the HCP modified with LS and NS against efflorescence, sulfate attack, calcium leaching, and so on, would be investigated in further research.

## Figures and Tables

**Table 1:** The formulation of cement pastes modified with sodium laurate (SL) and nano silica (NS).

Specimen	Mass (g)				Content (%)	Content (%)	CA (°)
	Cement	Water	SL	NS	SL	NS	
HCP	450	180	0	0	0	0%	0
HCPSL0.25	448.875	180	1.125	0	0.25	0%	109.5
HCPSL0.5	447.75	180	2.25	0	0.5%	0%	115.6
HCPSL0.75	446.625	180	3.375	0	0.75%	0%	129.5
HCPSL1.0	445.5	180	4.5	0	1.0%	0%	138.5
HCPSL1.25	444.375	180	5.625	0	1.25%	0%	141.7
HCPNS0.5	447.75	180	0	2.25	0%	0.5%	0
HCPNS1.0	445.5	180	0	4.5	0%	1.0%	0
HCPNS1.5	443.25	180	0	6.75	0%	1.5%	0
HCPSL0.25NS	444.375	180	1.125	4.5	0.25%	1.0%	105.6
HCPSL0.5NS	443.25	180	2.25	4.5	0.5%	1.0%	112.1
HCPSL0.75NS	442.125	180	3.375	4.5	0.75%	1.0%	121.3
HCPSL1.0 NS	441	180	4.5	4.5	1.0%	1.0%	131.2
HCPSL1.25NS	439.875	180	5.625	4.5	1.25%	1.0%	136.7

**Table 2:** Summary of water absorption and capillary water adsorption coefficient of samples.

Samples	HCP	HCPSL1.0	HCPNS1.0	HCPSL1.0NS1.0
Water absorption (%)	9.93	0.75	8.60	0.36
Capillary water adsorption coefficient (kg/m^2^·h^0.5^)	0.6602	0.0513	0.7345	0.0228
